# Validity and reliability of an adapted arabic version of the long international physical activity questionnaire

**DOI:** 10.1186/s12889-017-4599-7

**Published:** 2017-07-24

**Authors:** Khalil Helou, Nour El Helou, Maya Mahfouz, Yara Mahfouz, Pascale Salameh, Mireille Harmouche-Karaki

**Affiliations:** 1 Department of Nutrition, Faculty of Pharmacy, Saint Joseph University, B.P. 11-5076 Riad el Solh Beyrouth 1107 2180 Liban, Beirut, Lebanon; 2 Clinical and Epidemiological Research Laboratory, Faculty of Pharmacy, Lebanese University, Hadath, Lebanon

**Keywords:** International physical activity questionnaire, Arabic IPAQ, Physical activity, Reliability, Validity

## Abstract

**Background:**

The International Physical Actvity Questionnaire (IPAQ) is a validated tool for physical activity assessment used in many countries however no Arabic version of the long-form of this questionnaire exists to this date. Hence, the aim of this study was to cross-culturally adapt and validate an Arabic version of the long International Physical Activity Questionnaire (AIPAQ) equivalent to the French version (F-IPAQ) in a Lebanese population.

**Methods:**

The guidelines for cross-cultural adaptation provided by the World Health Organization and the International Physical Activity Questionnaire committee were followed. One hundred fifty-nine students and staff members from Saint Joseph University of Beirut were randomly recruited to participate in the study. Items of the A-IPAQ were compared to those from the F-IPAQ for concurrent validity using Spearman’s correlation coefficient. Content validity of the questionnaire was assessed using factor analysis for the A-IPAQ’s items. The physical activity indicators derived from the A-IPAQ were compared with the body mass index (BMI) of the participants for construct validity. The instrument was also evaluated for internal consistency reliability using Cronbach’s alpha and Intraclass Correlation Coefficient (ICC). Finally, thirty-one participants were asked to complete the A-IPAQ on two occasions three weeks apart to examine its test–retest reliability. Bland-Altman analyses were performed to evaluate the extent of agreement between the two versions of the questionnaire and its repeated administrations.

**Results:**

A high correlation was observed between answers of the F-IPAQ and those of the A-IPAQ, with Spearman’s correlation coefficients ranging from 0.91 to 1.00 (*p* < 0.05). Bland-Altman analysis showed a high level of agreement between the two versions with all values scattered around the mean for total physical activity (mean difference = 5.3 min/week, 95% limits of agreement = −145.2 to 155.8). Negative correlations were observed between MET values and BMI, independent of age, gender or university campus. The A-IPAQ showed a high internal consistency reliability with Cronbach’s alpha ranging from 0.769–1.00 (*p* < 0.001) and intraclass correlation coefficient (ICC) ranging from 0.625–0.999 (*p* < 0.001), except for a moderate agreement with the moderate garden/yard activity (alpha = 0.682; ICC = 0.518; *p* < 0.001). The A-IPAQ had moderate-to-good test-retest reliability for most of its items (ICC ranging from 0.66–0.96; *p* < 0.001) and the Bland-Altman analysis showed a satisfactory agreement between the two administrations of the A-IPAQ for total physical activity (mean difference = 99.8 min/week, 95% limits of agreement = −1105.3; 1304.9) and total vigorous and moderate physical activity (mean difference = −29.7 min/week, 95% limits of agreement = −777.6; 718.2).

**Conclusion:**

The modified Arabic version of the IPAQ showed acceptable validity and reliability for the assessment of physical activity among Lebanese adults. More studies are necessary in the future to assess its validity compared to a gold-standard criterion measure.

**Electronic supplementary material:**

The online version of this article (doi:10.1186/s12889-017-4599-7) contains supplementary material, which is available to authorized users.

## Background

Physical activity (PA) is effective in preventing numerous lifestyle-related chronic diseases, such as cardiovascular diseases, diabetes, hypertension [1]. In order to reduce the risk of non-communicable diseases and improve fitness and endurance levels, the World Health Organization (WHO) recommends practicing at least 150 min of moderate-intensity or 75 min of vigorous-intensity aerobic PA throughout the week, or an equivalent combination of both, for people aged 18 to 64 years [2]. PA is defined as regularly practiced exercises and includes all types of body movements [3] divided into 4 domains: occupational (related to work), domestic (house chores), transportation (walking, public transportation) and leisure time (recreational activities) [4].

Various objective and subjective methods are used for PA assessment. Objective methods consist of wearable monitors that measure bio signals such as heart rate or other indicators such as energy expenditure; they include indirect calorimetry, heart rate monitoring and motion sensors [4]. Subjective methods include PA diaries and questionnaires [4], and they are the most broadly adopted monitoring tools in multinational studies [5]. One example, the International Physical Activity Questionnaire (IPAQ), was conceived by an international consensus group in 1998 for young to middle-aged adults [5–7]. It exists in two forms, short and long, with a reference period of either “the last seven days” or “the usual week” [5, 6]. The IPAQ long form provides specific details on PA intensity levels in the four domains mentioned above and differentiates between usual sitting time on a week day and a weekend day [8]. It is a validated tool for PA assessment used in many countries [7] however no Arabic version of the long-form of this questionnaire exists to this date. Hence, the aim of this research was the cultural adaptation of the IPAQ to the Lebanese population. The future purpose will be to promote its use in the Arabic speaking countries (in the Middle East and North Africa region) and culturally adapt it to the said populations. Therefore, in this study, we aimed to develop an Arabic version of the IPAQ long form (A-IPAQ) equivalent to the validated French version, culturally adapt it to the target population and assess its validity and reproducibility on a sample of university students and staff members in Saint Joseph University (Université Saint Joseph – USJ), a Francophone university in Beirut, Lebanon.

## Methods

We used the method for cross-cultural adaptation recommended by the WHO and the IPAQ committee which consists of a forward translation of the questionnaire followed by a back translation [9, 10]. Subsequently, a pre-test was performed along with cognitive debriefing, before the final testing of the questionnaire on the final sample [9, 10].

### Forward translation into Arabic

The French version of the IPAQ long form (F-IPAQ) was first forward translated to Arabic by a single bilingual translator, familiar with the concepts included in the IPAQ. His mother-tongue language is Arabic and he is fluent in French. During this phase, the main focus was to achieve semantic equivalence between the French and Arabic versions while adopting a translation vocabulary easily comprehensible. The translated questionnaire was then reviewed by an expert committee to verify the idiomatic and conceptual equivalence of the Arabic translated version. The expert committee consisted of the original translator, healthcare professionals, an expert in physical activity and a language professional [11–14].

### Back translation into French

The Arabic version of the IPAQ was then blindly back-translated into the French language by a native French speaker translator, fluent in Arabic and unfamiliar with the concepts of the IPAQ and the original French version [11, 12]. The back-translated French questionnaire was subsequently compared to the original French one, by the expert committee, aiming to discern discrepancies and to solve any inconsistencies between the two versions. The process of forward-back translation was repeated until all ambiguities disappeared [10–17].

### Pre-testing

The pre-final A-IPAQ was then tested on a representative sample (status and sex distribution proportional to that of the target population per campus) composed of 40 bilingual volunteers, which is equivalent to 10 individuals per A-IPAQ section. The respondents completed both versions of the IPAQ, French and Arabic, during in-depth interviews. Simultaneously, cognitive debriefing was performed; the respondents were asked “probe questions” regarding what was meant by each question, the presence of any unusual, awkward, unclear or offending expressions, the necessity of additional activities or preferable alternatives, the rationale behind their choice and the acceptability of time limits [9, 10].

### Final testing

The final version of the A-IPAQ (see Additional file 1: for more details) was tested on a larger sample of 159 participants (10 participants per item) [14], representative of the target population, randomly selected from the students and staff database. The study sample was drawn using a stratified random cluster sampling by campus (medical sciences, human sciences, sports and innovation, social sciences and sciences and technologies). Recruitment efforts targeted a sample with a status and sex distribution proportional to that of the university population per campus. Both versions of the questionnaire were administered consecutively during the same interview and the order of administration (Arabic or French first) was randomly selected. Face-to-face interviews were adopted to ensure a higher response rate and avoid omission of particular questions. During the interview, data were collected regarding age, gender, marital status, occupational and financial status. The latter was assessed by a question asking the participants to describe their socioeconomic status (wealthy, moderately at ease or low). At this stage, the questionnaire was tested for validity and internal consistency reliability. Physical activity is negatively associated with overweight and obesity [1]. Hence, construct validity was determined using body mass index (BMI), as done in previous studies [18, 19]. Weight and height were measured and the BMI was calculated using the formula: weight (kg)/height(m)^2. Participants were considered overweight or obese if the BMI value ranged between 25 and 29.9 kg/m^2^ and ≥30 kg/m^2^ respectively [20]. Thirty-one participants agreed to complete the A-IPAQ a second time by the same interviewer in order to assess the test-retest reliability of the questionnaire. This sample size is generally considered sufficient to work with parametric tests in case of normal distribution (which is the case) [21] and a similar sample size has been used in other studies [22, 23]. This subgroup is considered representative of the original sample since gender, age and status distributions were similar in both samples. In agreement with other studies, the time frame between the first and the second administration was 3 weeks [24, 25]. We chose this period because it is long enough for the participants to forget their previous responses, but too short for any considerable changes in physical activity to occur.

### Ethics approval and consent to participate

This study was approved by the Ethics Committee of “Saint Joseph University of Beirut” (USJ-2012-19). Participants were fully informed about the purpose and procedures of the study before reading and signing the informed consent form.

### Statistical analysis

Means and standard deviations were computed for quantitative data and frequencies and distributions for qualitative data. We compared the items of the A-IPAQ to those of the F-IPAQ for concurrent validity using Spearman’s correlation coefficient and we performed Bland-Altman analyses to evaluate the extent of agreement between the Arabic and the French versions of the questionnaire as well as the first and the second administration of the A-IPAQ. A factor analysis was conducted using a promax rotation since factors were correlated. The Kaiser-Meyer-Olkin KMO measure was calculated, along with the Bartlett’s test. Anti-image correlation and communalities were also evaluated. We assessed the construct validity of the tool using Spearman’s correlation coefficient between body mass index (BMI) and PA indicators derived from the A-IPAQ: total MET-min/week and MET-min/week for walking, moderate PA, vigorous PA, leisure PA, transportation PA, housework and house maintenance PA, occupational PA and total sitting time. Cronbach’s alpha and intraclass correlation coefficient (ICC) were used to evaluate the A-IPAQ’s internal consistency reliability: the individual items were compared to total physical activity. ICC was also used for the test-retest reliability analysis of the A-IPAQ (two-way mixed effects model) and ICC values were interpreted by a common classification used in studies assessing the reliability and the validity of adapted IPAQ versions [23, 26], considering that an ICC value above 0.75 indicates good reliability, a value between 0.50–0.75 indicates moderate reliability while lower values reflect poor reliability. Statistical analyses were performed using IBM SPSS (IBM SPSS Statistics for Windows, Version 20, IBM corp., Armonk, NY). Confidence interval of 95% was used for all tests with a *p* value <0.05.

## Results

### Descriptive results

A total of 159 individuals (31.4% males; 68.8% females) with a mean age of 33.1 years (SD 12.9) participated in the study. Their characteristics are described in Table 1. The mean BMI was 23.88 kg/m^2^ (SD = 3.91, min = 16.94, and max = 40.26). The mean of daily MET-minutes calculated through the IPAQ scale was 284.78 (SD = 358.50; min = 0 and max = 1929). Out of 159 participants, 49 were instructors (30.8%), 50 (31.4%) were employees and 60 (37.7%) were students. As for financial status, 54 (34%) considered themselves wealthy, 78 (49.1%) were moderately at ease, while 27 (17%) declared having a lower socioeconomic status.

**Table 1:** Characteristics of the study population (*n* = 159)

	Total sample
Age (years)	33.1 ± 12.9
Gender, n (%)	
Men	50 (31.4)
Women	109 (68.6)
Weight (kg)	67.1 ± 14.1
Height (cm)	167.2 ± 8.3
BMI (kg/m^2^)	23.9 ± 3.9
BMI categories, n (%)	
Underweight	7 (4.4)
Normal weight	95 (60.1)
Overweight	47 (29.7)
Obese	9 (5.7)
Daily MET-minutes^a^	1082.7 ± 3.7
Marital status, n (%)	
Single	100 (62.9)
Engaged, married	56 (35.2)
Divorced, separated, widowed	3 (1.9)
Occupational status, n (%)	
Instructors	49 (30.8)
Employee	50 (31.4)
Student	60 (37.7)
Financial status, n (%)	
Wealthy	54 (34)
Moderately at ease	78 (49.1)
Lower socioeconomic status	27 (17)

BMI, body mass index; MET, metabolic equivalent task

^a^Daily MET-minutes = Total physical activity MET-minutes/week divided by 7 = sum of (Total Work + Total Transport + Total Domestic and Garden + Total Leisure-Time MET-minutes/week scores) /7 [41]

Values are displayed as mean ± standard deviation or number and percentage of participants

### Concurrent validity

**Fig. 1:**
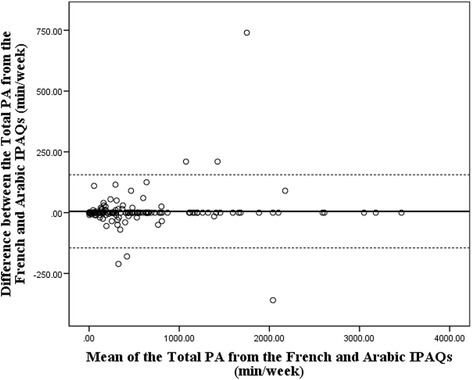
Bland Altman plot of the Total PA measured with the French and Arabic IPAQs. Mean difference = 5.3 min/week, 95% limits of agreement = −145.2 to 155.8

The results presented in Table 2 show a very high correlation between the Arabic and the French answers of bilingual individuals regarding all of the IPAQ’s items for job-related PA, transportation, housework and house maintenance, leisure activities as well as time spent sitting (“r” ranging from 0.91 to 1.00; *p* < 0.05). The Bland-Altman plot in Fig. 1 demonstrates a satisfactory agreement between the French and the Arabic IPAQ for total PA (mean difference = 5.3 min/week, 95% limits of agreement = −145.2;155.8). Furthermore, a regression analysis was performed and confirmed the absence of statistical bias (*p* > 0.05).

**Table 2:** Validity of the A-IPAQ versus the F-IPAQ using spearman’s correlation (*n* = 159)

PA Domain (minutes/week)	r
Job-related PA	
Vigorous PA	1.00**
Moderate PA	0.978**
Walking PA	0.999**
Transportation-related PA	
Motorized moving	0.92**
Bicycling	1.00*
Walking	0.988**
Housework, house maintenance-related PA	
Garden/yard vigorous PA	1.00*
Garden/yard moderate PA	0.91**
In-house moderate PA	0.997**
Leisure-related PA	
Leisure walks	0.98**
Vigorous leisure PA	0.945**
Moderate leisure PA	1.00**
Time spent sitting	
Total time spent sitting	0.981**
Daily time spent sitting (minutes/day)	0.989**
Time spent sitting on a weekend (minutes/day)	0.945**

**p < 0.001

*p < 0.05

A-IPAQ, Arabic International Physical Activity Questionnaire; F-IPAQ, French International Physical Activity Questionnaire; PA, physical activity

### Content validity

Factor analysis was performed after removing the item concerning moderate PA in the garden due to low communality with other items, and moderate in-house PA for inadequate loading. A moderate Kaiser-Meyer-Olkin (KMO) measure was found (0.507), with an adequate Bartlett’s test of Sphericity (*p* < 0.001). Anti-image correlation values were adequate. The total variance explained was 67.4%, spread on six factors: 14.8% for factor 1, 13.3% for factor 2, 11.7% for factor 3, 10.8% for factor 4, 8.9% for factor 5, and 7.8% for factor 6. Since factors were correlated, a Promax rotation was conducted and gave satisfactory results (see Additional file 2: Table S1 for more details).

### Construct validity

Globally, negative correlations were found between MET values and BMI, independent of age, gender or university campus. MET for housework and house maintenance activities for the total population was negatively correlated with BMI (*r* = −0.24; *p* < 0.01). In the male sample, BMI was negatively correlated with MET for total PA, moderate PA, and total leisure PA (*r* = −0.321, −0.393, −0.362, respectively; *p* < 0.05) whereas it was positively correlated with total sitting time in the female sample (*r* = 0.212; *p* < 0.05). Stratification by age also showed similar correlations for MET for total moderate PA and MET for housework and house maintenance activities among participants aged 30 years or less (*r* = −0.229, −0.325, respectively; *p* < 0.05) and for MET for total vigorous PA for those aged more than 30 years (*r* = −0.236; *p* < 0.05). The highest negative correlations with BMI were obtained among those studying at the Campus of Innovation and Sports with the MET for total moderate PA (*r* = −0.893; *p* < 0.01), and then among those studying at the Campus of Sciences and Technologies, with the MET for total PA, total vigorous PA, total walking and total leisure PA (*r* = −0.646, −0.754, −0.788, −0.611, respectively; *p* < 0.05). A negative correlation was observed with MET for housework and house maintenance activities with the Medical Sciences Campus (*r* = −0.301; *p* < 0.05) and a positive correlation between BMI and MET for total PA with the Campus of Social Sciences (*r* = 0.554; *p* < 0.05).

### Internal consistency reliability

Cronbach’s alpha and ICC of subscales and total scale are shown in Table 3. High agreement was found for all the weekly MET of the IPAQ’s items (Cronbach’s alpha ranging between 0.769 and 1.00; *p* < 0.001; and ICC ranging between 0.625 and 0.999; *p* < 0.001), except for a moderate agreement obtained with the MET for moderate garden/yard activity (alpha = 0.682; ICC = 0.518; *p* < 0.001).

**Table 3:** A-IPAQ reliability using Cronbach’s alpha and Intraclass Correlation Coefficient (ICC) (*n* = 159)

A-IPAQ measure (MET.minutes/week)	Cronbach’s alpha	ICC	95%CI
Total MET by PA type	0.994*	0.988*	0.984–0.991
Total MET by PA intensity	0.994*	0.988*	0.984–0.991
Daily MET-minutes^a^	0.99*	0.981*	0.974–0.986
PA level	0.997*	0.994*	0.992–0.996
Total vigorous PA	0.976*	0.954*	0.937–0.966
Total moderate PA	0.994*	0.988*	0.984–0.991
Total walking	0.998*	0.997*	0.995–0.998
Job-related PA	0.995*	0.99*	0.986–0.993
Vigorous PA	0.769*	0.625*	0.52–0.711
Moderate PA	1.00*	0.999*	0.999–0.999
Walking	0.998*	0.996*	0.995–0.997
Transportation-related PA	0.964*	0.931*	0.907–0.949
Walking	0.964*	0.93*	0.905–0.948
Bicycling	0.998*	0.996*	0.994–0.997
Housework, House Maintenance-related PA	0.989*	0.978*	0.97–0.984
Garden/yard vigorous activities	-	-	-
Garden/yard moderate activities	0.682*	0.518*	0.394–0.623
In-house moderate activities	0.999*	0.998*	0.997–0.998
Leisure-related PA	0.994*	0.987*	0.983–0.991
Leisure walks	0.988*	0.976*	0.967–0.982
Vigorous leisure PA	0.99*	0.979*	0.972–0.985
Moderate leisure PA	0.999*	0.998*	0.998–0.999

*p < 0.001

^a^Daily MET-minutes = Total physical activity MET-minutes/week divided by 7 = sum of (Total Work + Total Transport + Total Domestic and Garden + Total Leisure-Time MET-minutes/week scores) /7 [41]

A-IPAQ, Arabic International Physical Activity Questionnaire; MET, metabolic equivalent task; ICC, Intraclass Correlation Coefficient; PA, physical activity

### Test-retest reliability

Good reliability was shown between test and retest for the total vigorous PA and total moderate PA (ICC = 0.96 and 0.88 respectively) (Table 4). The job-related PA exhibited an ICC of 0.709 reflecting a moderate to good reliability. As for walking PA and total PA, reliability was moderate (ICC = 0.688 and 0.66 respectively), while it was poor for leisure-related PA and total sitting time (ICC = 0.493 and 0.414). An Additional file 3: Figure S2 shows the Bland-Altman plots of the duration of total PA (mean difference = 99.8 min/week, 95% limits of agreement = −1105.3; 1304.9) and total moderate-vigorous PA (mean difference = −29.7 min/week, 95% limits of agreement = −777.6; 718.2). Most data points were clustered around the line zero or the mean difference line across the range of METs. Few data points fell above the upper limit of agreement.

**Table 4:** Test-retest reliability of the A-IPAQ (*n* = 31)

A-IPAQ measure (MET.minutes/week)	First administration Mean (SD)	Second administration Mean (SD)	Intraclass correlation coefficient (ICC) (95% CI)	*p* value
Total PA	2570.4 (3017.0)	2310.6 (3255.1)	0.66 (0.404–0.82)	< 0.001
Total vigorous	449.2 (774.3)	671.3 (1035.7)	0.96 (−0.263–0.431)	< 0.001
Total moderate	971.3 (1959.8)	927.8 (2262.1)	0.88 (0.767–0.94)	< 0.001
Total walking	1158.5 (1983.9)	713.1 (1666.3)	0.688 (0.446–0.836)	< 0.001
Job-related PA	1515.4 (3033.6)	1240.7 (3079.0)	0.709 (0.478–0.848)	< 0.001
Transportation-related PA	124.5 (220.6)	99.8 (127.5)	0.543 (0.239–0.75)	0.001
Housework, House maintenance-related PA	219.7 (506.8)	349.2 (774.6)	0.081 (−0.276–0.419)	0.329
Leisure-related PA	710.8 (801.3)	620.8 (976.8)	0.493 (0.174–0.719)	0.002
Total time spent sitting (minutes/week)	3532.8 (1253.4)	3336.0 (1313.9)	0.414 (0.076–0.667)	0.009

A-IPAQ, Arabic International Physical Activity Questionnaire; SD, standard deviation; MET, metabolic equivalent task; CI, confidence interval; PA, physical activity

## Discussion

This study is the first to cross-culturally validate the IPAQ-long form to the Arabic language. We followed the cross-cultural adaptation method recommended by the WHO and the IPAQ committee which consisted of a forward translation of the long form F-IPAQ to Arabic, followed by a back-translation. This method is cost-efficient and widely applied in small budget studies which main concern is to establish source-target equivalence [15, 27], as in the present case. Subsequently, the pre-final version of the A-IPAQ was pre-tested on 40 individuals as recommended in the IPAQ guidelines where an ideal sample should comprise 30–40 respondents [10–12]. Testing the IPAQ on bilingual individuals, allowed the researchers to detect discrepancies between the two versions of the IPAQ [16, 28–30] and to perform “cognitive debriefing” in the context of in-depth interviews, that consisted in asking “probe questions” to the respondents to point out any unclear or offending sentences [9–12, 14, 27, 31, 32]. The last step consisted of testing the final A-IPAQ, on a sample size of 159 that matched the recommended range of 150–200 subjects, as in a minimum of 10 subjects per item [14].

We evaluated some aspects of validity and reliability of the Arabic IPAQ, as endorsed in the literature [27]. Very high correlation coefficients were obtained between the Arabic and the French versions of the IPAQ, indicating a satisfactory agreement between the two versions, and thus, very good concurrent validity. To evaluate the content validity of all items of the questionnaires we conducted KMO which was close to 0.6 suggesting a minimum value for a factor analysis. Bartlett’s test of sphericity was significant (*p* < 0.05) showing strength of intercorrelation between items of the questionnaire. Regarding communality, all items shared a total variance of 67.4%. The retained factors explained some of the variance present in the data. At last, Promax rotation showed a high correlation between all items of the questionnaire. The BMI was negatively associated with physical activity overall and most subscales as documented in the literature [33, 34] showing that the measure has some construct validity. The study results also showed significantly high ICC values for all IPAQ answers reflecting excellent reliability, except for moderate garden/yard activity which had a moderate agreement. There are many possible reasons for this latter observation; the type of residence of the majority of the participants (apartments without gardens) [35], or the students’ lack of interest in gardening, or the presence of gardeners specifically tasked with these duties. The mean Total PA observed for the first and second administration of the A-IPAQ were close, reaching 2570.4 (3017.0) and 2310.6 (3255.1) respectively. The study findings indicated moderate-to-good repeatability across the IPAQ domains, except for leisure-related PA, total sitting time, and housework/house maintenance-related PA. With the exception of the latter domain, all coefficients varied between 0.414 and 0.96, consistently with a previously reported range of values [7]. The highest test-retest reliability was found for the total vigorous PA similarly to previous studies [18, 22, 26], suggesting that exercises of high intensity are the most consistently practiced in comparison with lighter ones. Other domains that revealed comparable results to previous studies were the total moderate PA (ICC = 0.74 [22] and 0.823 [18] versus 0.88 in the present study) and the job-related PA (ICC ranging between 0.77 and 0.95 [18, 22, 23, 26] versus 0.709). Total PA and total walking PA demonstrated a moderate agreement that has been reported previously [23, 26]. Similarly, transportation-related PA also demonstrated a moderate repeatability (0.543) which could be explained by the inconsistent use of active transportation (bicycle or walking from home to the workplace), in favor of the excessive use of private cars by the majority of the Lebanese population [36]. A previous study conducted in Lebanon showed that participants who owned at least one car were less likely to be active than their counterparts with no car [37]. Leisure-related PA and total sitting time demonstrated a poor repeatability, most likely due to variability in the behavior of the participants, rather than to variability in the reproducibility of the IPAQ itself. While the present study revealed the absence of any agreement with the housework/house maintenance-related PA, the domestic PA in other studies achieved the lowest corresponding ICC coefficients in comparison to the rest of the PA domains [22, 26]. This lack of agreement may be attributable to the irregular practice of these tasks, due to the common presence of domestic helpers in Lebanese houses [38].

### Strengths and limitations

To our knowledge, this study is the first to cross-culturally validate the long-form IPAQ to the Arabic language. We determined a high correlation between answers of the French and Arabic IPAQs, a high internal consistency reliability of the A-IPAQ, a moderate-to-good test-retest reliability for most of the IPAQ items, as well as significant negative correlations with the BMI in the construct validity analysis. One limitation of the study is that the criterion validity of the tool was not evaluated in this study. This type of validity indicates if the instrument is correlated to a “gold-standard measure” of physical activity such as activity monitors [14, 39, 40]. Studies on the validity of the A-IPAQ compared to a gold-standard criterion measure should be done in the future. Another limitation is the KMO value that is slightly lower than the recommended 0.6 for factor analysis. Also, the reproducibility analysis was performed on 31 participants only, which may limit the generalizability of the results. However, although our sample may not be representative of the general Lebanese population, it covered a wide range of age, occupational status and included individuals from different Lebanese regions that attend Saint Joseph University. Further evaluation of the questionnaire is needed in various age groups and occupations, as well as in different populations of the Arab speaking countries.

## Conclusion

The present study shows that the French and Arabic versions of the IPAQ are equivalent to use in the Lebanese bilingual adult population. Based on this, the Arabic version has acceptable validity and reliability for the assessment of physical activity among Lebanese adults. More studies are necessary in the future to assess its validity compared to a gold-standard criterion measure.

## Additional files

Additional file 1: Questionnaire. Arabic version of the IPAQ developed for this study. (PDF 340 kb)

Additional file 2: Table S1. Content validity of the A-IPAQ. (PDF 12 kb)

Additional file 3: Figure S2. Bland-Altman plots of the duration of total PA and total moderate-vigorous PA determined on the first and the second administrations of the A-IPAQ. (PDF 208 kb)

## Abbreviations

A-IPAQ: Arabic international physical activity questionnaire
BMI: Body mass index
F-IPAQ: French international physical activity questionnaire
ICC: Intraclass correlation coefficient
IPAQ: International physical activity questionnaire
KMO: Kaiser-Meyer-Olkin
MET: Metabolic equivalent task
NEAT: Non-exercise activity thermogenesis
PA: Physical activity
USJ: Université Saint-Joseph (Saint Joseph University)
WHO: World Health Organization
