# Supplementary material for: Validity and reliability of an adapted arabic version of the long international physical activity questionnaire
Source: BMC Public Health. 2017 Jul 24;18:49. doi: 10.1186/s12889-017-4599-7 (PMC5525276; doi:10.1186/s12889-017-4599-7)
Supplement: Supplementary file 1 — Questionnaire. Arabic version of the IPAQ developed for this study. (PDF 340 kb) [file 12889_2017_4599_MOESM1_ESM.pdf]

# استبانة النشاطات البدنية خلال الأيام السبعة الأخيرة

---

**إقرأ على المشارك:** سوف أسألك بعض الأسئلة عن الوقت الذي قمت فيه بأنشطة بدنية خلال الأيام السبعة الماضية. لطفاً أجب عن كل سؤال من الأسئلة التالية حتى وإن كنت تعتبر نفسك غير نشيط بدنياً. فكر في الأنشطة البدنية التي تمارسها خلال عملك، وكجزء من أعمالك المنزلية، و أثناء تنقلك من مكان لآخر، و تلك التي تقوم بها في وقت فراغك بغرض الترفيه أو التمرين أو الرياضة.

## الجزء الأول: النشاطات البدنية المرتبطة بالعمل

**إقرأ على المشارك:** إن الأسئلة الأولى مرتبطة بعملك أي بالوظيفة أو العمل الذي تقوم به مقابل أجر أو مجاناً ويشمل ذلك على سبيل المثال لا الحصر الوظيفة المكتبية والعمل الزراعي والخدمة المجانية والتعليم والتحصيل العلمي ومتابعة تمرين وظيفي أو مهني (Stage) وأي نوع آخر من العمل مقابل أجر أو مجاناً خارج المنزل. لا تأخذ بعين الإعتبار العمل المجاني الذي تقوم به في المنزل مثل التنظيف والترتيب والعمل في الحديقة وأعمال الصيانة المنزلية والإهتمام بأفراد عائلتك أو المقيمين معك لأنني سأقوم بطرح الأسئلة عليك في هذا الخصوص لاحقاً.

**1- هل تقوم بعمل أو بوظيفة مقابل أجر أو مجاناً خارج المنزل؟ (عمل: نعم = 1، لا = 0؛ 8، 9 )**

**____ نعم**

**____ لا (إنتقل إلى الجزء الثاني)**

**8. لا يدري/ أو غير متأكد (إنتقل إلى الجزء الثاني)**

**9. رفض الإجابة**

(**إيضاح من مجري المقابلة:** إن العمل يشمل كما ذكرنا تحصيل العلم والتعليم ومتابعة تمرين وظيفي أو مهني (Stage) والعمل مجاناً مثل الخدمات الإجتماعية كما يشمل البحث عن عمل ولكنه لا يشمل الأعمال التي تقوم بها في منزلك وفي حديقتك والإهتمام بأفراد عائلتك أو المقيمين معك لأن ذلك سيكون موضوع أسئلة لاحقة.)

**إقرأ على المشارك:** إن الأسئلة التالية تشمل كافة النشاطات البدنية التي قمت بها خلال عملك أكان بدل أجر أو مجاناً ولكن ذلك لا يشمل التنقلات بين المنزل ومركز العمل.

**إقرأ على المشارك:** الآن فكّر في جميع الأنشطة البدنية التي تتطلّب جهداً بدنياً مرتفع الشدة والتي قمت بممارستها خلال الأيام السبعة الماضية. الأنشطة البدنية المرتفعة الشدة هي تلك الأنشطة التي تجعل تنفّسك أعلى بكثير من المعتاد، مثل رفع أشياء ثقيلة، أو حراثة الأرض، أو أعمال التعمير أو صعود السلالم (الأدراج). فكر فقط في الأنشطة البدنية المرتفعة الشدة التي قمت بممارستها لمدة 10 دقائق متواصلة على الأقل في كل مرة.

**2- خلال الأيام السبعة الماضية، كم يوماً مارست فيه نشاطاً بدنياً مرتفع الشدة ضمن العمل؟ (من 0 إلى 7، 8، 9)**

**____ يوم في الأسبوع (إذا أجاب المشارك صفر إنتقل إلى السؤال الرابع)**

**8. لا يدري/ أو غير متأكد (إنتقل إلى السؤال الرابع)**

**9. رفض الإجابة أو لم يجب (إنتقل إلى السؤال الرابع)**

(**إيضاح من مجري المقابلة:** فكّر فقط في الأنشطة البدنية المرتفعة الشدة التي قمت بممارستها لمدة 10 دقائق متواصلة على الأقل في كل مرة).

(**إيضاح من مجري المقابلة:** إن العمل يشمل الوظائف بدل أجر و/أو مجاناً والتعليم وتحصيل العلم والتمارين المهنية والوظيفية (Stage) كما يشمل العمل المجاني والبحث عن عمل).

**3- في المعتاد، كم من الوقت مارست فيه نشاط بدني مرتفع الشدة ضمن العمل كل يوم ؟**

**___ ___ ساعة في النـهار (من 0 إلى 16)**

**___ ___ دقيقة في النـهار (من 0 إلى 960، 998، 999)**

**998. لا يدري/ أو غير متأكد**

**999. رفض الإجابة أو لم يجب**

(**إيضاح من مجري المقابلة:** فكر فقط في الأنشطة البدنية مرتفعة الشدة التي قمت بممارستها لمدة 10 دقائق متواصلة على الأقل في كل مرة).

(**إيضاح من مجري المقابلة:** نحاول هنا تحديد معدّل يومي للوقت (متوسط الوقت اليومي) المخصّص للنشاط البدني المرتفع الشدة أما إذا كان المشارك لا يستطيع الإجابة لأن الوقت المخصّص للنشاط البدني المرتفع الشدة يتفاوت من يوم إلى آخر أو لأن طبيعة الأعمال التي يقوم بها متنوعة جداً ويصعب تحديد معدّل يومي لها فعندها يجب طرح السؤال كالآتي : **"ما هو الوقت الإجمالي (مجموع الوقت) الذي مارست فيه نشاط بدني مرتفع الشدة ضمن العمل خلال الأيام السبعة الأخيرة؟")**

**___ ___ ساعة في الأسبوع (من 0 إلى 112)**

**___ ___ ___ ___ دقيقة في الأسبوع (من 0 إلى 6720، 9998، 9999)**

**9998. لا يدري/ أو غير متأكد**

**9999. رفض الإجابة أو لم يجب**

**إقرأ على المشارك:** الآن فكّر في جميع الأنشطة البدنية التي تتطلب جهداً بدنياً معتدل الشدة والتي قمت بممارستها خلال الأيام السبعة الماضية. الأنشطة البدنية المعتدلة الشدة هي تلك الأنشطة التي تجعل تنفسك أعلى من المعتاد إلى حدٍّ ما، ويمكن أن تتضمن رفع أشياء خفيفة. لا تحسب المشي ضمن هذه الأنشطة. مرة أخرى، فكّر فقط في الأنشطة البدنية المعتدلة الشدة التي قمت بممارستها لمدة 10 دقائق متواصلة على الأقل في كل مرة.

**4- خلال الأيام السبعة الماضية، كم يوماً مارست فيه نشاطاً بدنياً معتدل الشدة ضمن العمل (من 0 إلى 7، 8، 9)؟**

**____ يوم في الأسبوع (إذا كان الجواب 0 إنتقل إلى السؤال رقم 6)**

**8. لا يدري/ أو غير متأكد (إنتقل إلى الجواب رقم 6)**

**9. رفض الإجابة أو لم يجب (إنتقل إلى الجواب رقم 6)**

(**إيضاح من مجري المقابلة:** فكر فقط في الأنشطة البدنية المعتدلة الشدة التي قمت بممارستها لمدة 10 دقائق متواصلة على الأقل في كل مرة.)

(**إيضاح من مجري المقابلة:** إن العمل يشمل الوظائف بدل أجر و/أو مجاناً والدرس وتحصيل العلم والتمارين المهنية والوظيفية (Stage) كما يشمل العمل المجاني والبحث عن عمل.)

**5- في المعتاد، كم من الوقت مارست فيه نشاط بدني معتدل الشدة ضمن العمل كل يوم ؟**

**___ ___ ساعة في النـهار (من 0 إلى 16)**

**___ ___ دقيقة في النـهار (من 0 إلى 960، 998، 999)**

**998. لا يدري/ أو غير متأكد**

**999. رفض الإجابة أو لم يجب**

(**إيضاح من مجري المقابلة:** فكر فقط في الأنشطة البدنية المعتدلة الشدة التي قمت بممارستها لمدة 10 دقائق متواصلة على الأقل في كل مرة.)

**(إيضاح من مجري المقابلة:** نحاول هنا تحديد معدل وسطي يومي للوقت (متوسط الوقت اليومي) المخصص للنشاط البدني المعتدل الشدة أما إذا كان المشارك لا يستطيع الإجابة لأن الوقت المخصص للنشاط البدني المعتدل الشدة يتفاوت من يوم إلى آخر أو لأن طبيعة الأعمال التي يقوم بها متنوعة جداً ويصعب تحديد معدل يومي لها فعندها يجب طرح السؤال كالآتي : **"ما هو الوقت الإجمالي (مجموع الوقت) الذي مارست فيه نشاط بدني معتدل الشدة ضمن العمل خلال الأيام السبعة الأخيرة؟")**

**___ ___ ساعة في الأسبوع (من 0 إلى 112)**

**___ ___ ___ ___ دقيقة في الأسبوع (من 0 إلى 6720، 9998، 9999)**

**9998. لا يدري/ أو غير متأكد**

**9999. رفض الإجابة أو لم يجب**

**إقرأ على المشارك:** الآن فكر في الوقت الذي مشيت فيه على الأقدام ضمن العمل على الأقل لمدة 10 دقائق متواصلة خلال الأيام السبع الماضية. لا يتضمن ذلك المشي من المنزل إلى العمل وبالعكس.

**6- خلال الأيام السبعة الماضية، كم يوماً مارست فيه المشي على الأقدام ضمن العمل لمدة 10 دقائق على الأقل في كل مرة؟ (من 0 إلى 7، 8، 9)**

**____ يوم في الأسبوع (إذا كان الجواب 0 إنتقل إلى الجزء الثاني)**

**8. لا يدري/ أو غير متأكد (إنتقل إلى الجزء الثاني)**

**9. رفض الإجابة أو لم يجب (إنتقل إلى الجزء الثاني)**

**(إيضاح من مجري المقابلة:** فكر فقط في المشي على الأقدام الذي قمت به ضمن العمل لمدة 10 دقائق متواصلة على الأقل في كل مرة.**)**

**(ملحوظة لمجري المقابلة:** خذ بعين الإعتبار كافة أنواع العمل.**)**

**7- في المعتاد، كم من الوقت مارست فيه المشي على الأقدام ضمن العمل كل يوم ؟**

**___ ___ ساعة في اليوم (من 0 إلى 16)**

**___ ___ ___ دقيقة في اليوم (من 0 إلى 960، 998، 999)**

**998. لا يدري/ أو غير متأكد**

**999. رفض الإجابة أو لم يجب**

**(إيضاح من مجري المقابلة:** فكر فقط في المشي على الأقدام الذي قمت به ضمن العمل لمدة 10 دقائق متواصلة على الأقل في كل مرة.**)**

**(متابعة من مجري المقابلة:** المطلوب هو معدل وسطي (متوسط) للوقت المخصص للمشي على الأقدام ضمن العمل لأحد الأيام. إذا لم يتمكن المجيب من الإجابة عن السؤال لأن وقت المشي على الأقدام تفاوت كثيراً من يوم لآخر، أسأله: **كم مجموع الوقت الذي مارست فيه المشي على الأقدام ضمن العمل خلال الأيام السبعة الماضية ؟)**

**___ ___ ساعة في الأسبوع (من 0 إلى 112)**

**___ ___ ___ ___ دقيقة في الأسبوع (من 0 إلى 6720، 9998، 9999)**

**9998. لا يدري/ أو غير متأكد**

**9999. رفض الإجابة أو لم يجب**

## الجزء الثاني: النشاطات البدنية المرتبطة بالتنقلات

**إقرأ على المشارك:** الآن فكر في الطريقة التي تنقلت فيها من مكان إلى آخر وتحديداً للذهاب إلى العمل أو إلى المحلات التجارية أو إلى السينما أو إلى أي مكان آخر.

**8- خلال الأيام السبعة الماضية، كم يوماً تنقلت فيه بوسيلة نقل آلية مثل السيارة والباص والدراجة النارية والقطار؟ (من 0 إلى 7، 8، 9)**

**____ يوم في الأسبوع (إذا كان الجواب 0 إنتقل إلى السؤال رقم 10)**

**8. لا يدري/ أو غير متأكد (إنتقل إلى السؤال رقم 10)**

**9. رفض الإجابة (إنتقل إلى السؤال رقم 10)**

**9- في المعتاد، ما هو الوقت الذي تنقلت فيه بوسائل النقل الآلية (سيارة أو دراجة نارية أو باص أو قطار) في كل يوم؟**

**___ ___ ساعة في اليوم (من 0 إلى 16)**

**___ ___ ___ دقيقة في اليوم (من 0 إلى 960، 998، 999)**

**998. لا يدري/ أو غير متأكد**

**999. عدم الإجابة أو رفض الإجابة**

(**متابعة من مجري المقابلة:** المطلوب هو معدل وسطي (متوسط) للوقت الذي خصصته للتنقل بوسائل النقل الآلية لأحد الأيام. إذا لم يتمكن المجيب من الإجابة عن السؤال لأن الوقت المخصص للتنقل بالآليات تفاوت كثيراً من يوم لآخر، أسأله: **كم مجموع الوقت الذي تنقلت فيه بوسائل النقل الآلية خلال الأيام السبعة الماضية ؟)**

**___ ___ ساعة في الأسبوع (من 0 إلى 112)**

**___ ___ ___ ___ دقيقة في الأسبوع (من 0 إلى 6720، 9998، 9999)**

**9998. لا يدري/ أو غير متأكد**

**9999. عدم الإجابة أو رفض الإجابة**

**إقرأ على المشارك: الآن** فكر في كافة تنقلاتك بواسطة الدراجة الهوائية سواءً للتنقل بين المنزل ومكان العمل أو للذهاب للتسوق وشراء الحاجيات أو لأي غرض آخر ولا تأخذ بعين الإعتبار سوى المسافات التي تطلبت 10 دقائق متواصلة وما فوق.

**10- خلال الأيام السبعة الماضية، كم يوماً تنقلت فيه بواسطة الدراجة الهوائية من مكان إلى آخر؟ (من 0 إلى 7، 8، 9)**

**____ يوم في الأسبوع (إذا كان الجواب 0 إنتقل إلى السؤال رقم 12)**

**8. لا يدري/ أو غير متأكد (إنتقل إلى السؤال رقم 12)**

**9. رفض الإجابة (إنتقل إلى السؤال رقم 12)**

(**إيضاح من مجري المقابلة:** فكر فقط في إستعمال الدراجة الهوائية لمدة 10 دقائق متواصلة على الأقل في كل مرة.)

**11- في المعتاد، ما هو معدل الوقت الذي تنقلت فيه كل يوم بواسطة الدراجة الهوائية ؟**

**___ ___ ساعة في اليوم (من 0 إلى 16)**

**___ ___ ___ دقيقة في اليوم (من 0 إلى 960، 998، 999)**

**998. لا يدري/ أو غير متأكد**

**999. عدم الإجابة أو رفض الإجابة**

(**إيضاح من مجري المقابلة:** فكر فقط في إستعمال الدراجة الهوائية لمدة 10 دقائق متواصلة على الأقل في كل مرة.)

(**متابعة من مجري المقابلة:** المطلوب هو معدل وسطي (متوسط) للوقت المخصص لإستعمال الدراجة الهوائية لأحد الأيام. إذا لم يتمكن المجيب من الإجابة عن السؤال لأن الوقت المخصص للتنقل بالدراجة الهوائية تفاوت كثيراً من يوم لآخر، إسأله: **كم مجموع الوقت الذي تنقلت فيه بواسطة الدراجة الهوائية خلال الأيام السبعة الماضية ؟)**

**___ ___ ساعة في الأسبوع (من 0 إلى 112)**

**___ ___ ___ ___ دقيقة في الأسبوع (من 0 إلى 6720، 9998، 9999)**

**9998.   لا يدري/ أو غير متأكد**
**9999.   عدم الإجابة أو رفض الإجابة**

**إقرأ على المشارك:** الآن فكر في الوقت الذي تنقلت فيه مشياً على الأقدام بين المنزل ومكان العمل أو للذهاب للتسوق وشراء الحاجيات أو لأي غرض آخر ولا تأخذ بعين الإعتبار سوى المسافات التي تطلبت 10 دقائق متواصلة وما فوق كما لا تأخذ بعين الإعتبار المشي على الأقدام بهدف الترفيه أو الرياضة الذي لا يرمي إلى التنقل من مكان إلى آخر.

**12-  خلال الأيام السبعة الماضية، كم يوماً تنقلت فيه مشياً على الأقدام؟ (من 0 إلى 7، 8، 9)**

**____ يوم في الأسبوع (إذا كان الجواب 0 إنتقل إلى الجزء الثالث)**
**8.   لا يدري/ أو غير متأكد (إنتقل إلى الجزء الثالث)**
**9.   رفض الإجابة أو لم يجب (إنتقل إلى الجزء الثالث)**

(**إيضاح من مجري المقابلة:** فكر فقط في المشي على الأقدام الذي قمت به لمدة 10 دقائق متواصلة على الأقل في كل مرة.)

**13-  في المعتاد، ما هو الوقت الذي تنقلت فيه مشياً على الأقدام كل يوم ؟**

**___ ___ ساعة في اليوم (من 0 إلى 16)**
**___ ___ ___ دقيقة في اليوم (من 0 إلى 960، 998، 999)**
**998.   لا يدري/ أو غير متأكد**
**999.   رفض الإجابة أو لم يجب**

(**إيضاح من مجري المقابلة:** فكر فقط في المشي الذي قمت به لمدة 10 دقائق متواصلة على الأقل في كل مرة.)

(**متابعة من مجري المقابلة:** المطلوب هو متوسط الوقت الذي خصصته للمشي على الأقدام للتنقل لأحد الأيام. إذا لم يتمكن المجيب من الإجابة عن السؤال لأن وقت الممارسة تفاوت كثيراً من يوم لآخر، أسأله: **كم مجموع الوقت الذي تنقلت فيه مشياً على الأقدام خلال الأيام السبعة الماضية ؟)**

**___ ___ ساعة في الأسبوع (من 0 إلى 112)**
**___ ___ ___ ___ دقيقة في الأسبوع (من 0 إلى 6720، 9998، 9999)**
**9998.   لا يدري/ أو غير متأكد**
**9999.   رفض الإجابة أو لم يجب**

## الجزء الثالث: النشاطات المنزلية مثل تنظيف وترتيب وصيانة المنزل والإهتمام بأفراد العائلة

**إقرأ على المشارك:** الآن فكر في النشاطات البدنية التي قمت بها في داخل المنزل وحوله مثل أعمال التنظيف والترتيب والإهتمام بالحديقة المنزلية وأعمال الصيانة والإهتمام بأفراد العائلة وغيرهم من المقيمين معك في المنزل.

**إقرأ على المشارك:** الآن فكر بجميع الأنشطة البدنية التي تتطلب جهداً بدنياً مرتفع الشدة والتي قمت بممارستها في الحديقة أو في باحة المنزل. الأنشطة البدنية المرتفعة الشدة هي تلك الأنشطة التي تجعل تنفسك أعلى بكثير من المعتاد، مثل رفع أشياء ثقيلة، أو تقطيع الحطب أو الحفر بالرفش أو بالمعول. فكر فقط في الأنشطة البدنية المرتفعة الشدة التي قمت بممارستها لمدة 10 دقائق متواصلة على الأقل في كل مرة.

**14-  خلال الأيام السبعة الماضية، كم يوماً مارست فيه نشاطاً بدنياً مرتفع الشدة في حديقة أو باحة المنزل؟ (من 0 إلى 7، 8، 9)**

**____ يوم في الأسبوع (إذا أجاب المشارك صفر إنتقل إلى السؤال 16)**
**8.   لا يدري/ أو غير متأكد (إنتقل إلى السؤال 16)**

9. **رفض الإجابة أو لم يجب (إنتقل إلى السؤال 16)**

(**إيضاح من مجري المقابلة:** فكر فقط في الأنشطة البدنية المرتفعة الشدة التي قمت بممارستها لمدة 10 دقائق متواصلة على الأقل في كل مرة.)

**-15 في المعتاد، كم من الوقت مارست فيه نشاطاً بدنياً مرتفع الشدة في حديقة أو باحة منزلك كل يوم؟**

**___ ___ ساعة في النهار (من 0 إلى 16)**

**___ ___ دقيقة في النهار (من 0 إلى 960، 998، 999)**

**998. لا يدري/ أو غير متأكد**

**999. رفض الإجابة أو لم يجب**

(**إيضاح من مجري المقابلة:** فكر فقط في الأنشطة البدنية المرتفعة الشدة التي قمت بممارستها لمدة 10 دقائق متواصلة على الأقل في كل مرة.)

(**إيضاح من مجري المقابلة:** نحاول هنا تحديد معدل يومي للوقت (متوسط الوقت اليومي) المخصص للنشاط البدني المرتفع الشدة أما إذا كان المشارك لا يستطيع الإجابة لأن الوقت المخصص للنشاط البدني المرتفع الشدة يتفاوت من يوم إلى آخر أو لأن طبيعة الأعمال التي يقوم بها متنوعة جداً ويصعب تحديد معدل يومي لها فعندها يجب طرح السؤال كالآتي : **"ما هو الوقت الإجمالي (مجموع الوقت) الذي مارست فيه نشاطاً بدنياً مرتفع الشدة أثناء العمل في حديقة أو باحة منزلك خلال الأيام السبعة الأخيرة؟"**)

**___ ___ ساعة في الأسبوع (من 0 إلى 112)**

**___ ___ ___ ___ دقيقة في الأسبوع (من 0 إلى 6720، 9998، 9999)**

**9998. لا يدري/ أو غير متأكد**

**9999. رفض الإجابة أو لم يجب**

**إقرأ على المشارك:** الآن فكر بجميع الأنشطة البدنية التي تتطلب جهداً بدنياً معتدل الشدة والتي قمت بممارستها في الحديقة أو في باحة المنزل خلال الأيام السبعة الأخيرة. الأنشطة البدنية المعتدلة الشدة هي تلك الأنشطة التي تجعل تنفسك أعلى من المعتاد إلى حد ما، مثل رفع أشياء خفيفة، أو الكناسة وتنظيف زجاج النوافذ وترتيب المكتبة والعمل بمشط الحديقة. فكر فقط في الأنشطة البدنية المعتدلة الشدة التي قمت بممارستها لمدة 10 دقائق متواصلة على الأقل في كل مرة.

**-16 خلال الأيام السبعة الماضية، كم يوماً مارست فيه نشاطاً بدنياً معتدل الشدة في حديقة أو باحة المنزل؟ (من 0 إلى 7، 8، 9)**

**____ يوم في الأسبوع (إذا أجاب المشارك صفر إنتقل إلى السؤال 18)**

**8. لا يدري/ أو غير متأكد (إنتقل إلى السؤال 18)**

**9. رفض الإجابة أو لم يجب (إنتقل إلى السؤال 18)**

(**إيضاح من مجري المقابلة:** فكر فقط في الأنشطة البدنية المعتدلة الشدة التي قمت بممارستها لمدة 10 دقائق متواصلة على الأقل في كل مرة.)

**-17 في المعتاد، كم من الوقت مارست فيه نشاطاً بدنياً معتدل الشدة في حديقة أو باحة منزلك كل يوم؟**

**___ ___ ساعة في النهار (من 0 إلى 16)**

**___ ___ دقيقة في النهار (من 0 إلى 960، 998، 999)**

**998. لا يدري/ أو غير متأكد**

**999. رفض الإجابة أو لم يجب**

(**إيضاح من مجري المقابلة:** فكر فقط في الأنشطة البدنية المعتدلة الشدة التي قمت بممارستها لمدة 10 دقائق متواصلة على الأقل في كل مرة.)

(**إيضاح من مجري المقابلة:** نحاول هنا تحديد معدل يومي للوقت (متوسط الوقت اليومي) المخصص للنشاط البدني المعتدل الشدة في حديقة أو باحة المنزل أما إذا كان المشارك لا يستطيع الإجابة لأن الوقت المخصص للنشاط البدني المعتدل الشدة في حديقة أو باحة المنزل يتفاوت من يوم إلى آخر أو لأن طبيعة الأعمال التي يقوم بها متنوعة جداً ويصعب تحديد معدل يومي لها فعندها يجب طرح السؤال كالآتي : **"ما هو الوقت الإجمالي (مجموع الوقت) الذي مارست فيه نشاطاً بدنياً معتدل الشدة أثناء العمل في حديقة أو باحة منزلك خلال الأيام السبعة الأخيرة؟"**)

**___ ___ ساعة في الأسبوع (من 0 إلى 112)**

**___ ___ ___ ___ دقيقة في الأسبوع (من 0 إلى 6720، 9998، 9999)**

**9998. لا يدري/ أو غير متأكد**

**9999. رفض الإجابة أو لم يجب**

**إقرأ على المشارك:** الآن فكر في جميع الأنشطة البدنية التي تتطلب جهداً بدنياً معتدل الشدة والتي قمت بممارستها في داخل المنزل خلال الأيام السبعة الأخيرة. الأنشطة البدنية المعتدلة الشدة هي تلك الأنشطة التي تجعل تنفسك أعلى من المعتاد إلى حد ما، مثل رفع أشياء خفيفة، أو الكناسة وتنظيف زجاج النوافذ ومسح الأرض. فكر فقط في الأنشطة البدنية المعتدلة الشدة التي قمت بممارستها لمدة 10 دقائق متواصلة على الأقل في كل مرة.

**18- خلال الأيام السبعة الماضية، كم يوماً مارست فيه نشاطاً بدنياً معتدل الشدة في داخل المنزل؟ (من 0 إلى 7، 8، 9)**

**____ يوم في الأسبوع (إذا أجاب المشارك صفر إنتقل إلى الجزء الرابع)**

**8. لا يدري/ أو غير متأكد (إنتقل إلى الجزء الرابع)**

**9. رفض الإجابة أو لم يجب (إنتقل إلى الجزء الرابع)**

(**إيضاح من مجري المقابلة:** فكر فقط في الأنشطة البدنية معتدلة الشدة التي قمت بممارستها لمدة 10 دقائق متواصلة على الأقل في كل مرة.)

**19- في المعتاد، كم من الوقت مارست فيه نشاطاً بدنياً معتدل الشدة في داخل منزلك كل يوم ؟**

**___ ___ ساعة في النهار (من 0 إلى 16)**

**___ ___ دقيقة في النهار (من 0 إلى 960، 998، 999)**

**998. لا يدري/ أو غير متأكد**

**999. رفض الإجابة أو لم يجب**

(**إيضاح من مجري المقابلة:** فكر فقط في الأنشطة البدنية معتدلة الشدة التي قمت بممارستها لمدة 10 دقائق متواصلة على الأقل في كل مرة.)

(**إيضاح من مجري المقابلة:** نحاول هنا تحديد معدل يومي للوقت (متوسط الوقت اليومي) المخصص للنشاط البدني المعتدل الشدة داخل المنزل أما إذا كان المشارك لا يستطيع الإجابة لأن الوقت المخصص للنشاط البدني المعتدل الشدة داخل المنزل يتفاوت من يوم إلى آخر أو لأن طبيعة الأعمال التي يقوم بها متنوعة جداً ويصعب تحديد معدل يومي لها فعندها يجب طرح السؤال كالآتي : **"ما هو الوقت الإجمالي (مجموع الوقت) الذي مارست فيه نشاطاً بدنياً معتدل الشدة أثناء العمل في داخل منزلك خلال الأيام السبعة الأخيرة؟"**)

**___ ___ ساعة في الأسبوع (من 0 إلى 112)**

**___ ___ ___ ___ دقيقة في الأسبوع (من 0 إلى 6720، 9998، 9999)**

**9998. لا يدري/ أو غير متأكد**

**9999. رفض الإجابة أو لم يجب**

## الجزء الرابع: النشاطات البدنية المرتبطة بالترفيه والرياضة والوقت الحر

**أقرأ على المشارك:** فكر الآن بجميع الأنشطة البدنية التي مارستها خلال الأيام السبعة الأخيرة في إطار وقتك الحر ونشاطاتك الرياضية والترفيهية. لا تأخذ في عين الإعتبار كافة النشاطات البدنية التي ذكرتها سابقاً.

**20- عدا عن المشي على الأقدام الذي ذكرته سابقاً خلال الأيام السبعة الماضية، كم يوماً مارست فيه المشي على الأقدام لمدة 10 دقائق على الأقل خلال أوقات فراغك ؟ (من 0 إلى 7، 8، 9)**

**____ يوم في الأسبوع (إذا كان الجواب 0 إنتقل إلى السؤال 22)**

**8. لا يدري/ أو غير متأكد (إنتقل إلى السؤال 22)**

**9. رفض الإجابة أو لم يجب (إنتقل إلى السؤال 22)**

(**إيضاح من مجري المقابلة:** فكر فقط في المشي على الأقدام الذي قمت به لمدة 10 دقائق متواصلة على الأقل في كل مرة.)

**21- في المعتاد، ما هو الوقت الذي خصصته للمشي على الأقدام خلال أوقات فراغك كل يوم ؟**

**___ ___ ساعة في اليوم (من 0 إلى 16)**

**___ ___ ___ دقيقة في اليوم (من 0 إلى 960، 998، 999)**

**998. لا يدري/ أو غير متأكد**

**999. رفض الإجابة أو لم يجب**

(**إيضاح من مجري المقابلة:** فكر فقط في المشي الذي قمت به لمدة 10 دقائق متواصلة على الأقل في كل مرة.)

(**متابعة من مجري المقابلة:** المطلوب هو المعدل الوسطي (متوسط) للوقت الذي خصصته للمشي على الأقدام خلال أوقات الفراغ في اليوم الواحد. إذا لم يتمكن المجيب من الإجابة عن السؤال لأن وقت الممارسة تفاوت كثيراً من يوم لآخر، أسأله: **كم مجموع الوقت الذي مشيت فيه على الأقدام أثناء أوقات الفراغ خلال الأيام السبعة الماضية ؟**)

**___ ___ ساعة في الأسبوع (من 0 إلى 112)**

**___ ___ ___ ___ دقيقة في الأسبوع (من 0 إلى 6720، 9998، 9999)**

**9998. لا يدري/ أو غير متأكد**

**9999. رفض الإجابة أو لم يجب**

**إقرأ على المشارك:** الآن فكر بجميع الأنشطة البدنية الأخرى التي مارستها خلال الأيام السبعة الأخيرة في إطار وقتك الحر ونشاطاتك الرياضية والترفيهية لمدة 10 دقائق متواصلة على الأقل. لا تأخذ في عين الإعتبار كافة النشاطات البدنية التي ذكرتها سابقاً.

**إقرأ على المشارك:** الآن فكر في جميع الأنشطة البدنية التي تتطلب جهداً بدنياً مرتفع الشدة والتي قمت بممارستها في أوقات فراغك خلال الأيام السبعة الأخيرة. الأنشطة البدنية المرتفعة الشدة هي تلك الأنشطة التي تجعل تنفسك أعلى بكثير من المعتاد مثل الجري (الركض) وركوب الدراجة الهوائية والسباحة السريعة والرياضة الهوائية (Aérobie). فكر فقط في الأنشطة البدنية مرتفعة الشدة التي قمت بممارستها لمدة 10 دقائق متواصلة على الأقل في كل مرة.

**22- خلال الأيام السبعة الماضية كم يوماً مارست فيه نشاطات بدنية مرتفعة الشدة خلال أوقات فراغك لمدة 10 دقائق على الأقل ؟ (من 0 إلى 7، 8، 9)**

**____ يوم في الأسبوع (إذا كان الجواب 0 إنتقل إلى السؤال 24)**

**8. لا يدري/ أو غير متأكد (إنتقل إلى السؤال 24)**

**9. رفض الإجابة أو لم يجب (إنتقل إلى السؤال 24)**

23- **في المعتاد، ما هو الوقت الذي خصصته لممارسة نشاطات بدنية مرتفعة الشدة خلال أوقات فراغك كل يوم ؟**

**___ ___ ساعة في اليوم (من 0 إلى 16)**
**___ ___ ___ دقيقة في اليوم (من 0 إلى 960، 998، 999)**
**998. لا يدري/ أو غير متأكد**
**999. رفض الإجابة أو لم يجب**

(**إيضاح من مجري المقابلة:** فكر فقط في النشاط البدني المرتفع الشدة الذي قمت به خلال أوقات فراغك لمدة 10 دقائق متواصلة على الأقل في كل مرة.)

(**متابعة من مجري المقابلة:** المطلوب هو معدل وسطي (متوسط) للوقت الذي خصصته للنشاط البدني المرتفع الشدة خلال أوقات فراغك في اليوم الواحد. إذا لم يتمكن المجيب من الإجابة عن السؤال لأن وقت الممارسة تفاوت كثيراً من يوم لآخر، أسأله: **كم مجموع الوقت الذي مارست فيه نشاطات بدنية مرتفعة الشدة خلال أوقات فراغك خلال الأيام السبعة الماضية ؟)**

**___ ___ ساعة في الأسبوع (من 0 إلى 112)**
**___ ___ ___ ___ دقيقة في الأسبوع (من 0 إلى 6720، 9998، 9999)**
**9998. لا يدري/ أو غير متأكد**
**9999. رفض الإجابة أو لم يجب**

**إقرأ على المشارك:** فكر الآن بجميع الأنشطة البدنية التي تتطلب جهداً بدنياً معتدل الشدة والتي قمت بممارستها في أوقات فراغك. الأنشطة البدنية المعتدلة الشدة هي تلك الأنشطة التي تجعل تنفسك أعلى من المعتاد إلى حد ما مثل ركوب الدراجة الهوائية والسباحة الهادئة والرياضة الهوائية وكرة المضرب المزدوجة. فكر فقط في الأنشطة البدنية المعتدلة الشدة التي قمت بممارستها لمدة 10 دقائق متواصلة على الأقل في كل مرة.

24- **كم يوماً مارست فيه نشاطات بدنية معتدلة الشدة خلال أوقات فراغك لمدة 10 دقائق على الأقل ؟ (من 0 إلى 7، 8، 9)**

**____ يوم في الأسبوع (إذا كان الجواب 0 إنتقل إلى الجزء الخامس)**
**8. لا يدري/ أو غير متأكد (إنتقل إلى الجزء الخامس)**
**9. رفض الإجابة أو لم يجب (إنتقل إلى الجزء الخامس)**

25- **في المعتاد، ما هو الوقت الذي مارست فيه نشاطات بدنية معتدلة الشدة خلال أوقات فراغك كل يوم ؟**

**___ ___ ساعة في اليوم (من 0 إلى 16)**
**___ ___ ___ دقيقة في اليوم (من 0 إلى 960، 998، 999)**
**998. لا يدري/ أو غير متأكد**
**999. رفض الإجابة أو لم يجب**

(**إيضاح من مجري المقابلة:** فكر فقط في النشاط البدني المعتدل الشدة الذي قمت به خلال أوقات فراغك لمدة 10 دقائق متواصلة على الأقل في كل مرة.)

(**متابعة من مجري المقابلة:** المطلوب هو المعدل الوسطي (متوسط) للوقت الذي خصصته للنشاط البدني المعتدل الشدة خلال أوقات فراغك في اليوم الواحد. إذا لم يتمكن المجيب من الإجابة عن السؤال لأن وقت الممارسة تفاوت كثيراً من يوم لآخر، أسأله: **كم مجموع الوقت الذي مارست فيه نشاطات بدنية معتدلة الشدة خلال أوقات فراغك خلال الأيام السبعة الماضية ؟)**

**___ ___ ساعة في الأسبوع (من 0 إلى 112)**
**___ ___ ___ ___ دقيقة في الأسبوع (من 0 إلى 6720، 9998، 9999)**
**9998. لا يدري/ أو غير متأكد**
**9999. رفض الإجابة أو لم يجب**

## الجزء الخامس: الوقت الذي قضيته جالساً

**إقرأ على المشارك:** إن هذا السؤال يشمل كافة الأوقات التي قضيتها جالساً أو مستلقياً دون نوم خلال الأيام السبعة الأخيرة. يشمل ذلك الجلوس ضمن العمل أو في المكتب وفي المنزل وأثناء حضور الدروس التعليمية أو ممارسة التعليم وخلال زيارة الأصدقاء وأثناء القراءة ومشاهدة التلفاز والإستماع إلى الموسيقى وأثناء التمدد على الأريكة دون نوم وخلال الترفيه وغيره. لا يشمل ذلك الوقت الذي قضيته في الآليات والسيارات والذي ذكرته سابقاً.

**26- في المعتاد، ما هو الوقت الوسطي (المعدل) الذي قضيته جالساً في يوم واحد من أيام الأسبوع العادية خلال الأيام السبعة الأخيرة ؟**

**___ ___ ساعة في اليوم (من 0 إلى 16)**
**___ ___ ___ دقيقة في اليوم (من 0 إلى 960، 998، 999)**
**998. لا يدري/ أو غير متأكد**
**999. رفض الإجابة أو لم يجب**

**(إيضاح من مجري المقابلة:** أشمل في جوابك الوقت الذي قضيته مستلقياً ومستيقظاً دون أن تنام.**)**

**(متابعة من مجري المقابلة:** المطلوب هو المعدل الوسطي (متوسط) للوقت الذي قضيته جالساً في اليوم الواحد وإذا لم يتمكن المجيب من الإجابة عن السؤال لأن وقت الجلوس تفاوت كثيراً من يوم لآخر في بحر الأسبوع، أسأله: **كم مجموع الوقت الذي قضيته جالساً يوم الأربعاء الماضي ؟)**

**___ ___ ساعة يوم الأربعاء الماضي (من 0 إلى 112)**
**___ ___ ___ ___ دقيقة في الأسبوع (من 0 إلى 6720، 9998، 9999)**
**9998. لا يدري/ أو غير متأكد**
**9999. رفض الإجابة أو لم يجب**

**27- في المعتاد، ما هو المعدل الوسطي للوقت الذي قضيته جالساً في يوم واحد من أيام نهاية الأسبوع خلال الأيام السبعة الأخيرة ؟**

**___ ___ ساعة في اليوم (من 0 إلى 16)**
**___ ___ ___ دقيقة في اليوم (من 0 إلى 960، 998، 999)**
**998. لا يدري/ أو غير متأكد**
**999. رفض الإجابة أو لم يجب**

**(إيضاح من مجري المقابلة:** أشمل في جوابك الوقت الذي قضيته مستلقياً ومستيقظاً دون أن تنام.**)**

**(متابعة من مجري المقابلة:** المطلوب هو المعدل الوسطي (متوسط) للوقت الذي قضيته جالساً في اليوم الواحد من عطلة نهاية السبوع وإذا لم يتمكن المجيب من الإجابة عن السؤال لأن وقت الجلوس تفاوت كثيراً من يوم لآخر، أسأله: **كم مجموع الوقت الذي قضيته جالساً يوم السبت الماضي ؟)**

**___ ___ ساعة يوم السبت الماضي (من 0 إلى 112)**
**___ ___ ___ ___ دقيقة في الأسبوع (من 0 إلى 6720، 9998، 9999)**
**9998. لا يدري/ أو غير متأكد**
**9999. رفض الإجابة أو لم يجب**
