# Supplementary material for: Validity and reliability of an adapted arabic version of the long international physical activity questionnaire
Source: BMC Public Health. 2017 Jul 24;18:49. doi: 10.1186/s12889-017-4599-7 (PMC5525276; doi:10.1186/s12889-017-4599-7)
Supplement: Supplementary file 2 — Content validity of the A-IPAQ. (PDF 12 kb) [file 12889_2017_4599_MOESM2_ESM.pdf]

Table S1: Content validity of the A-IPAQ

Item	Communality	Factor loadings
**Factor 1: Job-related PA**		
Total duration of walking	0.795	0.897
Total duration of vigorous PA	0.754	0.860
**Factor 2: Leisure PA except walks**		
Total duration of bike movements	0.646	0.777
Total duration of moderate leisure PA	0.594	0.770
Total duration of vigorous leisure PA	0.524	0.615
**Factor 3: Indoor sedentarity and PA**		
Mean daily sitting last week end	0.721	0.870
Mean daily sitting last week days	0.709	0.631
Moderate in-house PA	0.573	-0.558
**Factor 4: Additional PA**		
Total moderate PA at work	0.616	0.766
Total walking to move from one place to another	0.680	0.753
Total vigorous PA garden/yard	0.525	0.578
**Factor 5: Total motorized movement**	0.742	0.858
**Factor 6: Total leisure walk**	0.880	0.949
