# Supplementary material for: Validity and reliability of an adapted arabic version of the long international physical activity questionnaire
Source: BMC Public Health. 2017 Jul 24;18:49. doi: 10.1186/s12889-017-4599-7 (PMC5525276; doi:10.1186/s12889-017-4599-7)
Supplement: Supplementary file 3 — Bland-Altman plots of the duration of total PA and total moderate-vigorous PA determined on the first and the second administrations of the A-IPAQ. (PDF 208 kb) [file 12889_2017_4599_MOESM3_ESM.pdf]

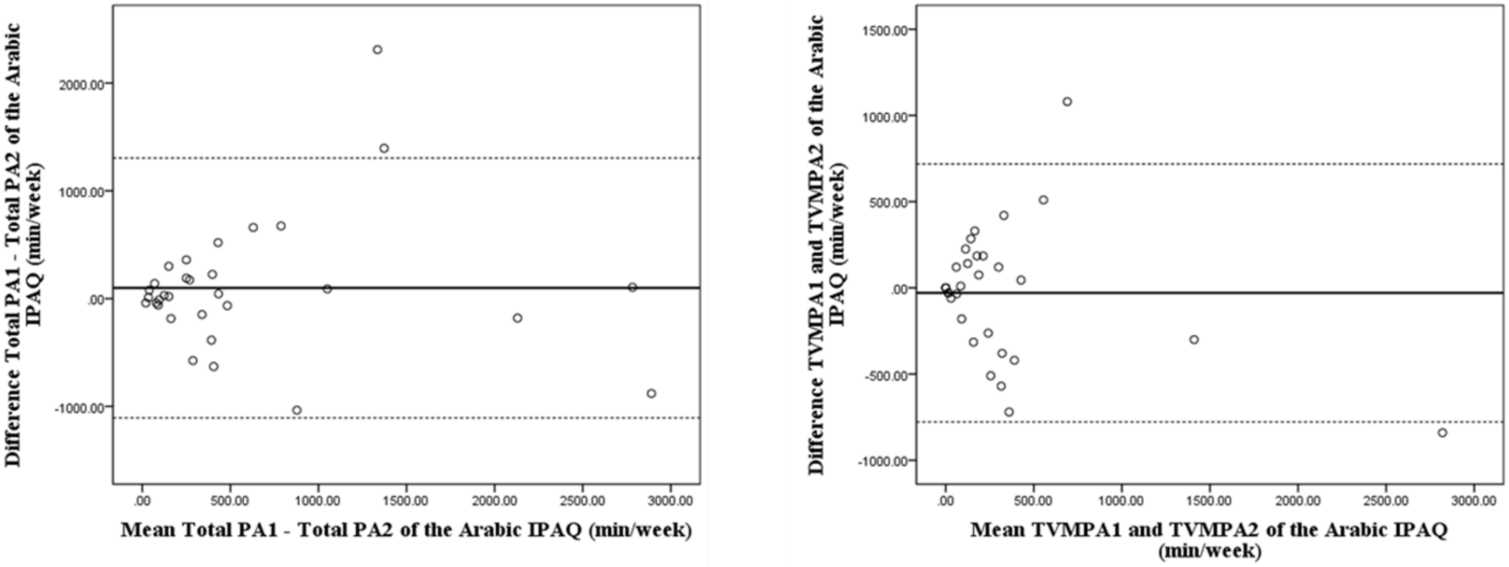

Figure S2:

2a: Bland-Altman plot of Total PA1 and Total PA2 determined on the first and the second administrations of the A-IPAQ (min/week). Mean difference = 99.8 min/week, 95% limits of agreement = -1105.3; 1304.9.

2b: Bland-Altman plot of Total Vigorous and Moderate PA1 and PA2 determined on the first and the second administrations of the A-IPAQ (min/week). Mean difference = -29.7 min/week, 95% limits of agreement = -777.6; 718.2.
